# A deep-learning approach for myocardial fibrosis detection in early contrast-enhanced cardiac CT images

**DOI:** 10.3389/fcvm.2023.1151705

**Published:** 2023-06-22

**Authors:** Marco Penso, Mario Babbaro, Sara Moccia, Andrea Baggiano, Maria Ludovica Carerj, Marco Guglielmo, Laura Fusini, Saima Mushtaq, Daniele Andreini, Mauro Pepi, Gianluca Pontone, Enrico G. Caiani

**Affiliations:** ^1^ Department of Perioperative Cardiology and Cardiovascular Imaging, Centro Cardiologico Monzino IRCCS, Milan, Italy; ^2^ Department of Electronics, Information and Biomedical Engineering, Politecnico di Milano, Milan, Italy; ^3^ Department of Cardiology, IRCCS Policlinico San Donato, Milan, Italy; ^4^ The BioRobotics Institute and Department of Excellence in Robotics and AI, Scuola Superiore Sant’Anna, Pisa, Italy; ^5^ Cardiovascular Section, Department of Clinical Sciences and Community Health, University of Milan, Milan, Italy; ^6^ Department of Biomedical Sciences and Morphological and Functional Imaging, “G. Martino” University Hospital Messina, Messina, Italy; ^7^ Department of Cardiology, Division of Heart and Lungs, Utrecht University, Utrecht University Medical Center, Utrecht, Netherlands; ^8^ Department of Cardiology, Haga Teaching Hospital, The Hague, Netherlands; ^9^ Department of Cardiology, Istituto Auxologico Italiano IRCCS, Milan, Italy

**Keywords:** cardiac computed tomography, delayed enhancement, artificial intelligence, scar tissue classification, deep learning, myocardial fibrosis

## Abstract

**Aims:**

Diagnosis of myocardial fibrosis is commonly performed with late gadolinium contrast-enhanced (CE) cardiac magnetic resonance (CMR), which might be contraindicated or unavailable. Coronary computed tomography (CCT) is emerging as an alternative to CMR. We sought to evaluate whether a deep learning (DL) model could allow identification of myocardial fibrosis from routine early CE-CCT images.

**Methods and results:**

Fifty consecutive patients with known left ventricular (LV) dysfunction (LVD) underwent both CE-CMR and (early and late) CE-CCT. According to the CE-CMR patterns, patients were classified as ischemic (*n* = 15, 30%) or non-ischemic (*n* = 35, 70%) LVD. Delayed enhancement regions were manually traced on late CE-CCT using CE-CMR as reference. On early CE-CCT images, the myocardial sectors were extracted according to AHA 16-segment model and labeled as with scar or not, based on the late CE-CCT manual tracing. A DL model was developed to classify each segment. A total of 44,187 LV segments were analyzed, resulting in accuracy of 71% and area under the ROC curve of 76% (95% CI: 72%−81%), while, with the bull’s eye segmental comparison of CE-CMR and respective early CE-CCT findings, an 89% agreement was achieved.

**Conclusions:**

DL on early CE-CCT acquisition may allow detection of LV sectors affected with myocardial fibrosis, thus without additional contrast-agent administration or radiational dose. Such tool might reduce the user interaction and visual inspection with benefit in both efforts and time.

## 1. Introduction

The presence and extent of myocardial fibrosis has a crucial prognostic and therapeutic role, potentially resulting in irreversible reduction in left ventricular (LV) function over time. Nowadays, gadolinium (Gd) contrast-enhanced (CE) cardiac magnetic resonance (CMR) imaging represents the gold-standard technique for the diagnosis and assessment of myocardial fibrosis (1). However, as the clinical use of CMR could be limited by resource availability, relative or absolute contraindications, and by clinical setting where CMR may not represent the first-line investigation (2, 3), an alternative reliable imaging technique to detect myocardial fibrosis would be highly desirable.

The CE-cardiac computed tomography (CCT) was recently demonstrated to be a potential accurate alternative to CMR for the identification of myocardial fibrosis (4). The AHA/ACC 2020 guidelines propose CCT as an alternative technique to evaluate myocardial properties (5), thus offering the possibility of combining non-invasive coronary evaluation with myocardial tissue characterization. Previous studies reported as late CE-CCT can be feasible and accurate for the detection of ischemic myocardial fibrosis when compared to CE-CMR (6, 7), providing similar performance in the assessment of myocardial viability in acute myocardial infarction (8, 9), even in non-ischemic fibrosis (7, 10). Despite these promising results, myocardial fibrosis assessment using late CE-CCT is limited by the low signal-to-noise-ratio and by the need of a higher dose of contrast and radiation when compared to coronary (i.e., early-enhancement) evaluation (11). However, in the early-enhancement phase, clinicians may fail to visually identify myocardial fibrosis.

The introduction in the clinical setting of artificial intelligence (AI) to process cardiac images has showed remarkable performance in both diagnosis and prognosis (12). We hypothesized that AI-based methodologies could help tackling the challenges relevant to myocardial fibrosis assessment using early CE-CCT, thus reducing the need of additional contrast and radiation dose, improving scar reading times and guiding clinical decision-making. Accordingly, we aimed at developing a deep-learning (DL) solution for the identification of the LV sectors affected with myocardial fibrosis with early CE-CCT images, and test its feasibility and accuracy using CE-CMR as reference.

## 2. Materials and methods

### 2.1. Study population

A consecutive cohort of fifty patients with an established diagnosis of LV dysfunction (LV ejection fraction <50%) undergoing CE-CMR between 2019 and 2020 were retrospectively selected from an Institutional resource program (13). CCT was performed per protocol within 10 days from CMR. Diagnosis of myocardial fibrosis from CMR was part of the inclusion criteria. Exclusion criteria included contraindications to contrast-agents or to CMR (such as pacemaker or claustrophobia) and impaired renal function (creatinine clearance <60 ml/min). The Institution’s ethical committee approved the protocol, and all patients gave written informed consent.

### 2.2. CMR protocol

As previously indicated (13), CMR was performed with a 1.5 T system (Discovery MR 450, GE Healthcare) using dedicated phased-array surface receiver coil and ECG triggering, CE breath-hold segmented T1-weighted inversion-recovery gradient-echo sequence for myocardial fibrosis was performed 10–20 min after an intravenous bolus of 0.1 mmol/kg of Gadobutrol (Gadovist, Bayer Schering Pharma AG). All images were acquired in LV short-axis (SAX) view. CE-CMR images, which represent the gold-standard reference, were investigated visually by one reader (EACVI Level III CMR certified reader). For each patient, based on the per-segment level, the presence of myocardial fibrosis based on the CE distribution was annotated according to the 17-segments American Heart Association (AHA) model. Diagnosis criteria for cardiomyopathy were associated with the CE pattern (see Supplementary Material for more details).

### 2.3. CCT protocol

All CE-CCT images were acquired using a Revolution CT (GE Healthcare) with slice configuration 256 × 0.625 mm, spatial resolution 0.230 mm along the X–Y planes, rotational speed time 280 ms and prospective ECG triggering. Scans were performed during the end-inspiratory phase using the breath-hold technique with patients in the supine position. Coronary images were acquired covering the entire cardiac cycle (R-R phases from 0% to 100%), after intravenous injection of 1.5 ml/kg of contrast medium (Iomeron 400 mg/ml), sub-divided into two boluses: 80 ml contrast medium through an antecubital vein at an infusion rate of 5 ml/s, followed by 50 ml saline solution and a second bolus of contrast medium to reach the predetermined total dose of contrast medium. Imaging was performed using the bolus tracking technique. A second series of ECG-gated breath-hold CE-CCT images was acquired for myocardial delayed enhancement after 8 min from the first contrast-agent injection (100 kVp; 400 mA). Late CE-CCT images were reconstructed at 75% of the R-R interval using soft kernel and 0.625 mm slice thickness. The effective dose was calculated as the dose-length product times a conversion coefficient for the chest (*K* = 0.014 mSv/mGy/cm) (14).

### 2.4. Data analysis and labelling

**Figure 1:**
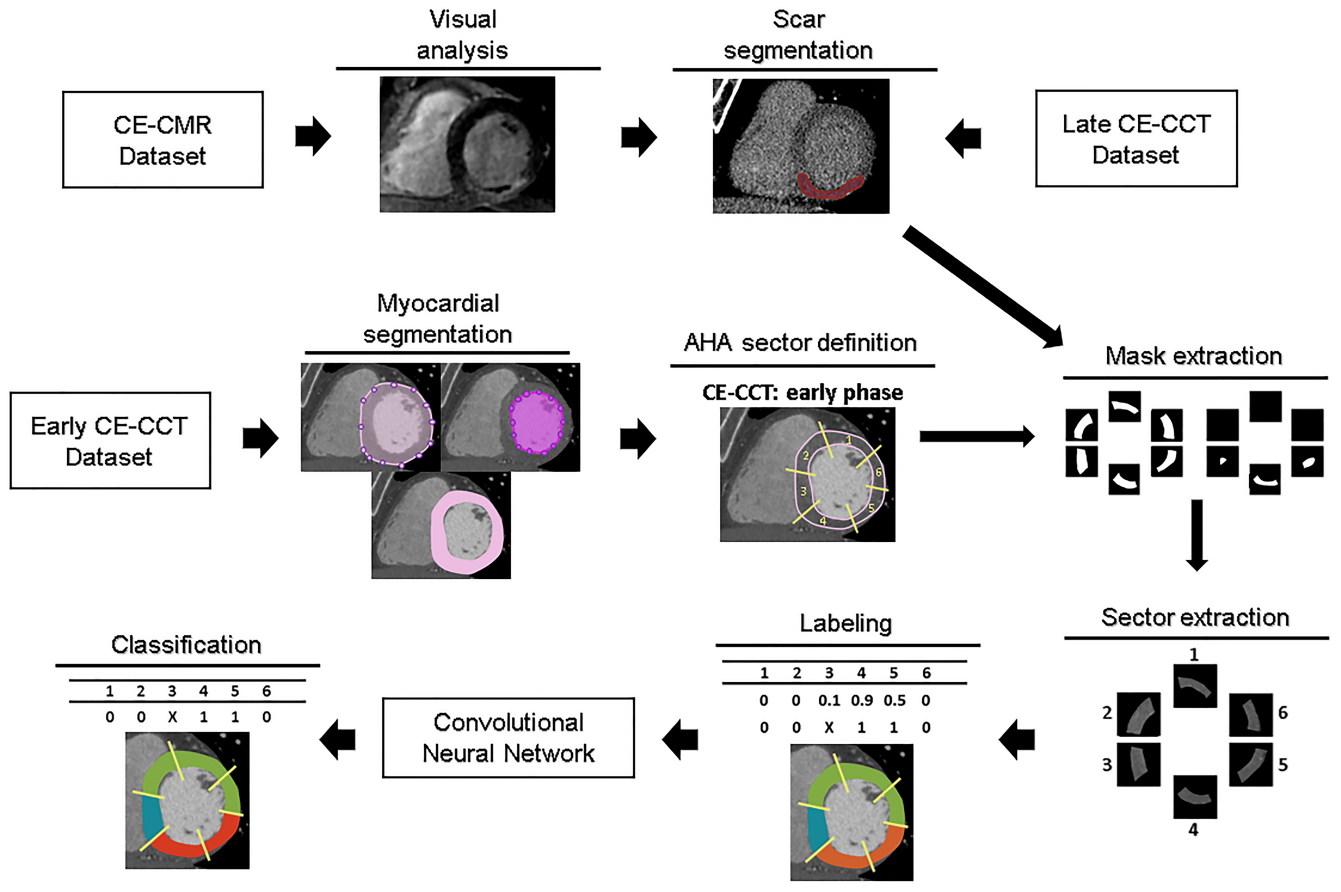
Workflow of the proposed approach for myocardial fibrosis detection. For training and testing purposes, for each early CE-CCT images the myocardial region was extracted and divided in sectors, according to the AHA model. Then scar ground-truths obtained with manual tracing of myocardial fibrosis contours on late CCT images were used to label each corresponding sector on early phase. Abbreviations as in Figure 1. AHA, American Heart Association; CCT, cardiac computed tomography; CMR, cardiac magnetic resonance; CE, contrast-enhanced.

**Figure 2:**
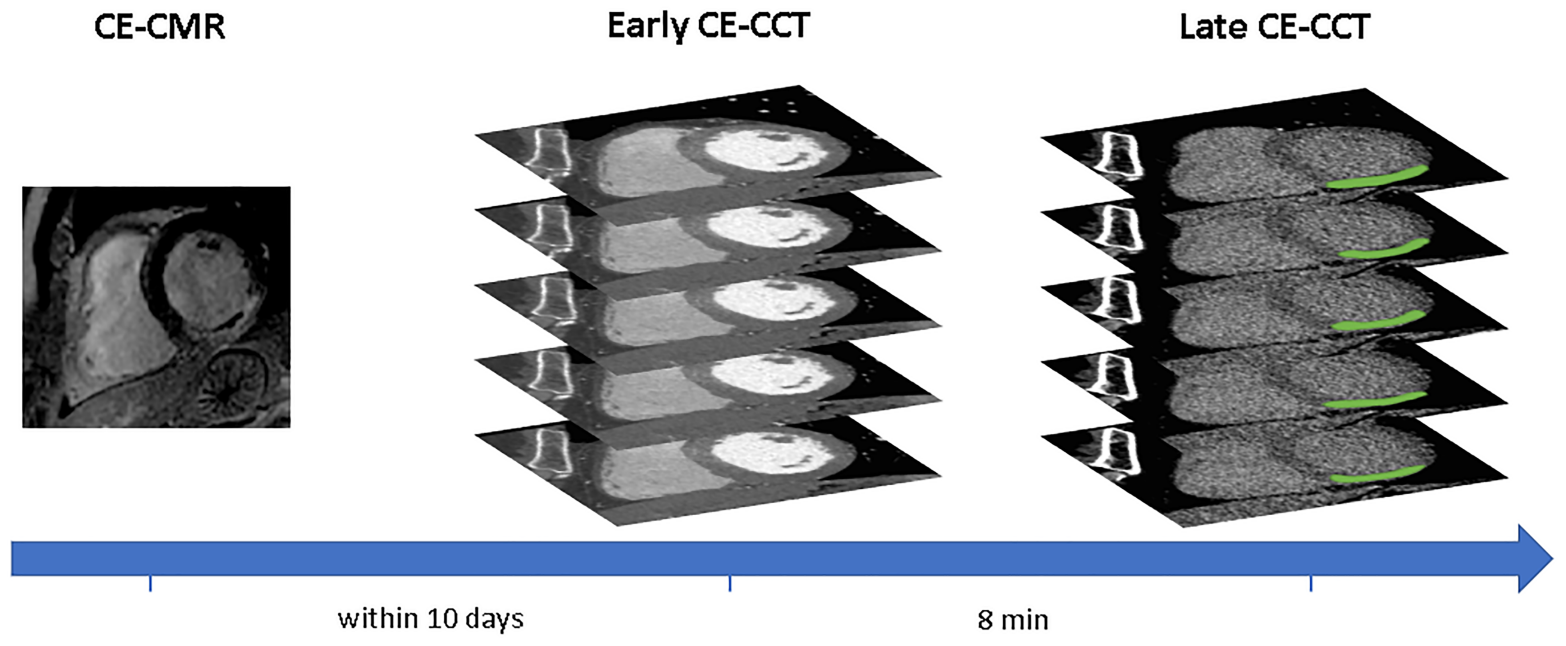
Example of hypoenhanced area on early and late contrast-enhanced CCT and corresponding contrast-enhanced CMR image. Abbreviations as in Figure 1.

A schematic illustration of the workflow is shown in Figure 1. Myocardial late CE (Figure 1, top) was evaluated on the SAX view from the base to the apex by two expert readers (EACVI guidelines for training and certification), after a proper optimization of the window settings. For each image, manual scar tracings was provided with the visual support of the corresponding CE-CMR distribution as reference, thus reducing regional disagreement between modalities. Each CE-CMR slice was automatically associated to multiple CE-CCT slices, based on its thickness and its position considering the total number of slices covering the entire LV in the two imaging techniques. An example of CE-CMR image (gold standard) and relevant associated ground-truth slices from late CE-CCT resulting from scar manual tracing is shown in Figure 2, together with the corresponding early CE-CCT.

In the early CE-CCT volume (Figure 1, middle), the myocardial (LV epicardial and endocardial) boundaries for each SAX cut-plane covering the whole heart were automatically segmented to delimit the scar searching area, by active contours region growing algorithm (15) and manually adjusted when needed (MATLAB®, The Mathworks). Papillary muscles were included in the cardiac blood pool as recommended (16). Based on the identified myocardial boundaries, a binary mask was obtained and multiplied with the original image, to retain only the videointensity information of the pixels in the myocardium for further processing with DL.

Considering the number of slices included between the LV base and apex, the LV obtained from the early CE-CCT was divided into 3 sections (basal, mid, apical) of equal length perpendicular to its long axis, thus generating three groups (basal, mid-cavity and apical) of SAX slices for the LV. For the basal section, only slices where the myocardium surrounds completely the LV were further considered for analysis. The apical cap was excluded from the apical section as recommended (17) (Supplementary Figure S1). Then, the corresponding LV myocardial region in each slice was divided into four (i.e., for apical) or six sectors, depending on the slice section (18), according to the 16-segment AHA model. For each sector, a reference label was attributed (Figure 1, bottom) based on the results of the scar manual tracing in the corresponding late CE-CCT image (i.e., in the same spatial location and at the same cardiac phase): the area of the traced scar for each sector was calculated, together with the area of the whole sector. A sector was labelled as “scar” if the segmented enhanced area occupied >15% of the sector area, and as “no scar” otherwise (see Supplementary Figure S2), as recommended (19). Moreover, this might potentially attenuate misalignment (e.g., different breath-holding) between early and late CE-CCT acquisitions.

### 2.5. Deep learning model training

For model training, each image corresponding to a myocardial sector in the early CE-CCT was cropped to reduce the processing area, and resized into 85 × 85 pixels. In addition, videointensity values in Hounsfield units were normalized to zero mean and unit variance.

To obtain the binary classification of myocardial tissue in scar/no scar, early CE-CCT sectors constituted the input of an end-to-end DL classification model based on a custom 2D-Convolutional Neural Network (CNN), together with the provided classification. The CNN was built within the DL framework Tensorflow-Keras (https://keras.io/) and consisted of 4 convolutional layers. The number of filters was set to 32, 64, 96 and 96, respectively. After each convolutional layer of kernel size 3 × 3, the feature volumes were down-sampled by a max-pooling layer with 2 × 2 pixels window. On top of the network, three fully-connected layers (256, 64 and 1 neurons, respectively) preceded the classification layer. After each max-pooling layer, batch normalization was implemented to make the training process faster and less sensitive to the learning rates. A random dropout of 30% was applied during training in each fully connected layer to prevent overfitting. The Rectified Linear Unit (ReLU) was used as activation function in all layers except the final one, where the sigmoid function was used. Training was performed from scratch using Adam optimizer with the initial learning rate set to 1 × 10^−3^ to minimize the binary cross-entropy loss function. The rate will be multiplied by a factor of 0.2 once the validation loss does not continuously reduce over 6 epochs, and the training phase will end when it reaches 1 × 10^−7^.

Model weights were initialized from normal distribution and the network was trained with L2 regularization with *λ* = 10 × 10^−3^ over 100 epochs on a batch size of 32 samples. All the training parameters were established with a trial-and-error procedure.

To augment the training data, data augmentation was created on the fly via randomly generated transforms, including rotation, scaling, flipping and translation. To deal with the intrinsic imbalanced nature of the dataset, a random undersampling strategy to the majority class (no scar label) was applied until a balanced ratio with the minority class (scar label) was reached.

### 2.6. Performance evaluation

To evaluate the classification performance, based on the ground truth evaluation built on the late CE-CCT images, a 5-fold cross-validation strategy (20) was performed (patient-wise) to reduce bias. For each of the 5 iterations in the validation, one-fold was used as the test dataset and the remaining four folds were used as the training dataset. The results of a per-segment analysis were evaluated using standard metrics: accuracy, positive predictive value (PPV), negative predictive value (NPV), sensitivity and Area Under the Curve (AUC) of the Receiver Operating Characteristic (ROC) curve (21). The final evaluation score was calculated by averaging the scores obtained in the 5 folds.

To compare this performance in a per-patient analysis with the CE-CMR gold standard, the 16-segment AHA model relevant to the presence/absence of a delayed enhancement in the early CE-CCT image was compared with the corresponding 16-segment CE-CMR model. More details about the AHA-sectors subdivision and analysis can be found in Supplementary Material. Moreover, every myocardial segment was evaluated separately and independently on late CE-CCT scan and compared with the proposed method.

Continuous variables were expressed as mean ± standard deviation (SD) or median (25th−75th percentiles), whereas categorical data were given as absolute value and percentage, as appropriate. Confidence intervals (CI) were set at 95%. Agreement between CCT and CMR in the number of myocardial segments involved was assessed with the Cohen K statistic.

## 3. Results

### 3.1. Population characteristics

Baseline characteristics of the study group (age 62 ± 10 years, 42 men) are reported in Table 1. According to CMR, 35 patients (70%) had a non-ischemic fibrosis (well-establish patterns associated to myocarditis, dilated cardiomyopathy, LV non-compaction or pathological hypertrophy), while 15 (30%) had a specific scar pattern corresponding to myocardial infarction. Mean effective doses were 7.7 ± 2.5 and 0.9 ± 0.3 mSv in the whole CCT and late phase, respectively.

**Table 1:** Patient characteristics.

	All (*n* = 50)
Age, years	62 ± 10
BMI, Kg/m^2^	26 ± 4
Female	8 (16%)
CMR	
LVEDV index (ml/m^2^)	118 (98–160)
LVESV index (ml/m^2^)	81 (57–113)
LVSV index (ml/m^2^)	41 (32–48)
LVEF (%)	33.7 ± 11.2
LV mass index (g/m^2^)	68 (57–88)
RVEDV index (ml/m^2^)	80 (61–97)
RVESV index (ml/m^2^)	37 (26–58)
RVEF (%)	50.3 ± 14.7
Ischemic	15 (30%)
Non-ischemic	35 (70%)
Number of coronary artery disease by CT	
0	33 (66%)
1	9 (18%)
2	3 (6%)
3	5 (10%)
Type of Cardiomyopathy	
Myocarditis	11 (22%)
Dilated	22 (44%)
LVNC	1 (2%)
Hypertrophic	1 (2%)

Values are mean ± SD, median (25th−75th percentiles) or *n* (%).

BMI, Body Mass Index; CMR, cardiac magnetic resonance; LV, left ventricular; EDV, end diastolic volume; ESV, end systolic volume; SV, stroke volume; EF, ejection fraction; RV, right ventricular; CT, computed tomography; NC, non-compaction.

According to early CE-CCT evaluation, 17 patients presented significant coronary artery disease: nine patients presented single-vessel disease, three had a two-vessel disease and 5 reported a triple vessel disease. For details on the segmental involvement and the respective CE-CMR pattern (subendocardial, mid-wall, subepicardial or transmural) see Supplementary Table S1.

### 3.2. Deep-learning classification

Of the initial 44,187 sectors computed out of the 8,285 slices available from the early CE-CCT images, 4,594 sectors (10%) presented scar, of which, according to the threshold condition based on the scar manual tracing, 1,090 were excluded, 3,504 (8%) were labeled as scar, and 39,593 (92%) were labelled as no scar.

**Figure 3:**
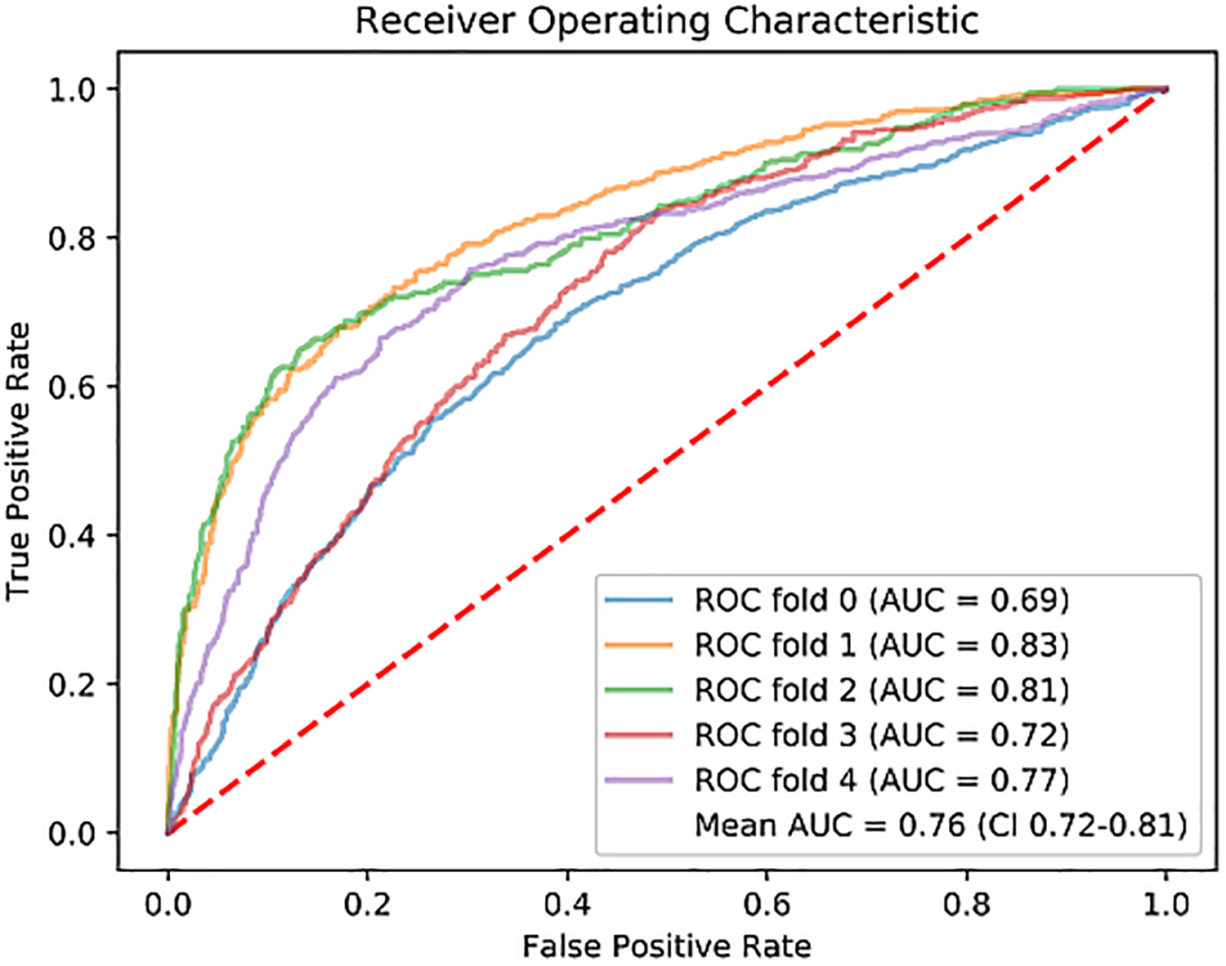
Receiver operating characteristic (ROC) curve for each fold of the 5-fold cross-validation, and the mean. ROC, Receiver Operating Characteristic; AUC, Area Under the Curve.

On the early CE-CCT images, a classification accuracy of 71% was obtained through the 5-fold cross validation (Table 2). The mean sensitivity, PPV and NPV for the testing fold resulted in 73%, 56% and 85%, respectively. Figure 3 shows the ROC curve obtained from all the tested folds. The mean AUC across the five folds was 76% (95% CI: 72%−81%). Diagnostic accuracy in ischemic, and non-ischemic cardiomyopathy patients was 66% and 74%, respectively, with AUC of 71% (95% CI: 64%−70%) and 80% (95% CI: 68%−93%), respectively (Table 2).

**Table 2:** Diagnostic accuracy of the model.

Patients	AUC (95%CI)	Accuracy	Sensitivity	PPV	NPV
All	0.76 (0.72–0.81)	0.71	0.73	0.56	0.85
Ischemic	0.71 (0.64–0.79)	0.66	0.64	0.56	0.75
Non-ischemic	0.80 (0.68–0.93)	0.74	0.77	0.56	0.88

AUC, area under the curve; CI, confidence interval; PPV, positive predictive value; NPV, negative predictive value.

**Figure 4:**
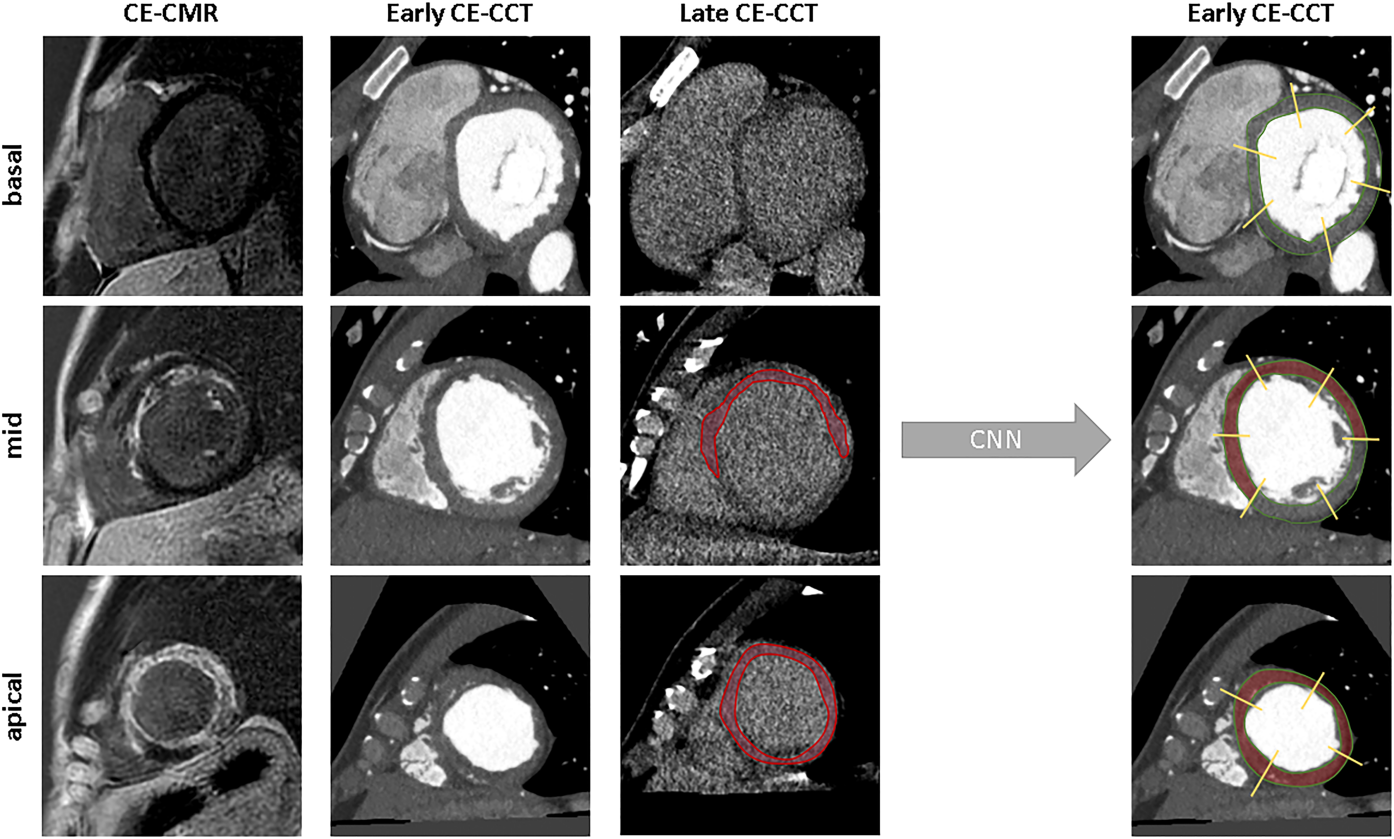
Examples of model’s classification for a basal, mid-ventricular and apical slice. From left to right: ground truth, raw image, scar segmentation and the predicted classification. CNN, convolutional neural network; other abbreviations as Figure 1.

Representative examples of the classification process for one slice at basal, mid and apical levels is given in Figure 4.

**Figure 5:**
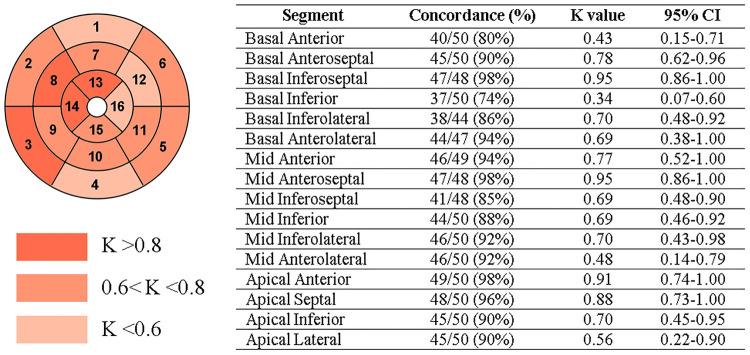
Per-segment analysis of the concordance (Cohen’s K statistic) between CE-CMR and CNN detection on early CE-CCT. CI: confidence interval.

In a per-segment analysis of the 16-segment AHA model, 708 out of 784 sectors were correctly classified, thus resulting in a total accuracy from the early CE-CCT of 89% (ischemic: 86%; non-ischemic: 92%) compared to the CE-CMR. The concordance between the model prediction and CMR assessment of CE extent is shown in Figure 5, with K values ranging between 0.34 (basal inferior segment) and 0.95 (basal inferoseptal segment). According to the CE-CMR model, 214 sectors were identified with fibrosis. Based on the DL-model prediction, 186 (87%, true positive) of these 214 sectors, and 522 (92%, true negative) of the 570 sectors with no-scar indication were correctly classified.

**Figure 6:**
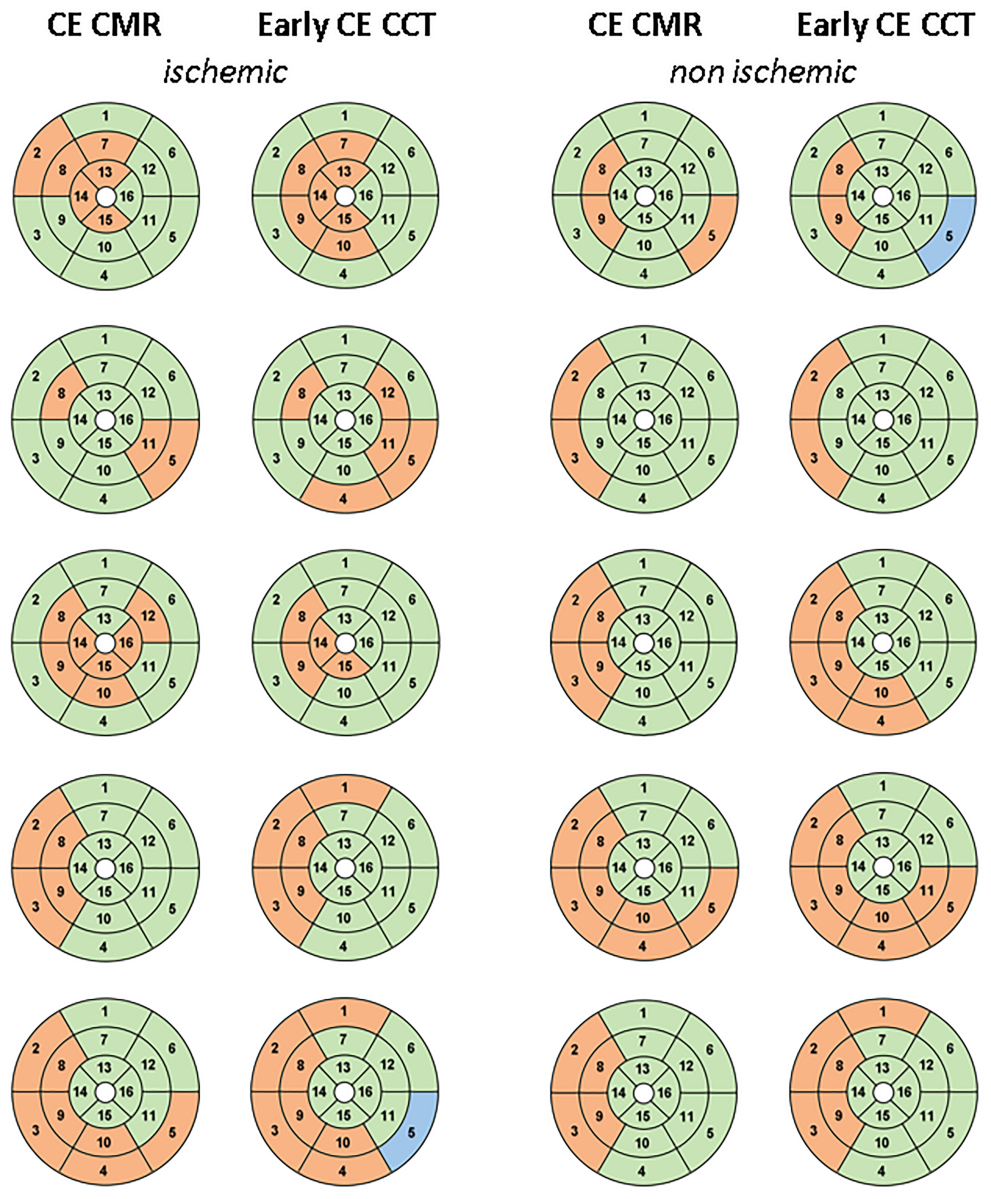
16-segment AHA model comparison between CE-CMR visual analysis and CNN detection on early CE-CCT for patients with ischemic and non-ischemic LV dysfunction: red and green represent pathological and healthy sectors, respectively. Blu indicates the sector excluded during pre-processing phase. Abbreviations as in Figure 1.

According to the visual analysis on late CE-CCT scan, the agreement with the CE-CMR was 82% (ischemic: 80%; non-ischemic: 84%). Results of the comparison between the 16-segment AHA model from the CE-CMR and that from the early CE-CCT, as determined by the CNN, is illustrated in Figure 6, for both ischemic and non-ischemic LV dysfunction patients, respectively, randomly selected from the whole population.

## 4. Discussion

An automatic AI system based on CNN for myocardial tissue classification from early CE-CCT imaging was developed and evaluated. Our results showed that its use in routinely noninvasive coronary imaging, without the need of a dedicated CCT acquisition, thus potentially eliminating the need for additional contrast-agent administration or radiation dose, enables myocardial fibrosis detection (AUC 0.76). Further, on a per-segment basis of the 16-segment model, the proposed method seemed to perform slightly better than the expert clinical myocardial fibrosis evaluation based on late CE-CCT scan (CNN accuracy 89% vs. 82% for expert). These results might pave the way towards a future approach for scar detection parallel to CMR, especially for those patients precluded from contrast-agent.

Besides the promising results, automatic sector classification did not reach always satisfactory results. A possible hypothesis could be that images of some patients were more critical to be classified than others. Accordingly, the effect of the classification accuracy for different patient classes (ischemic, non-ischemic) was examined. A lower performance in patients with an ischemic pattern over non-ischemic pattern was observed, in particular relevant to the decrease in NPV. Nevertheless, these findings may be of clinical relevance as automatic analysis of the 16-segment AHA model showed appreciable accuracy for both ischemic (86%) and non-ischemic (92%) cardiomyopathy patients, above the clinical observation accuracy on late CE-CCT (ischemic: 80%; non-ischemic: 84%).

Although CE-CMR imaging represents the reference technique for locating and qualifying myocardial fibrosis (22), it involves extending scanning time and the administration of Gd-based contrast-agent might be contraindicated. Advances in CCT imaging have led to its role as possible alternative technique to CMR, specifically for patients with poor access or contraindications and by its availability. Recently, CT scanners allow cardiac assessment without motion artefacts and elevated spatial resolution compared to CMR (11). Although exposure to ionizing radiations remains the main limitation factor for CCT, modern scanners showed accurate myocardium and coronary arteries diagnosis with appreciable image quality even using low-radiation dose (23, 24). On this regard, dual-energy CT and spectral CT may further improve CT image diagnostic quality (25), proving more detailed tissue information, thus advancing the potential utility of AI for myocardial fibrosis assessment.

Currently, the acquisition protocol for the delayed enhancement CCT imaging is based on injection of large volumes of iodinated contrast-agents (at least 1.5 ml/kg) which might cause different complications (9, 26), besides no complications were however observed in this study. Therefore, the implemented method might represent an attractive clinical solution, particularly to reduce user interaction and visual interpretation with benefit in both efforts and time, with only few seconds needed to automatically analyze the whole CCT volume. Furthermore, identification of myocardial fibrosis from CCT might improve strategy planning and prognosis even for patients precluded from CE-CMR or when transthoracic echocardiography is inconclusive.

So far, only few studies investigated how to detect myocardial fibrosis from routine coronary CCT using AI-based approaches. A first attempt exploiting DL for the identification of ischemic myocardial fibrosis from early CE-CCT was proposed in (27). However, scar analysis was performed on a limited number of 25 CCT datasets. In comparison, we proposed a larger and heterogeneous population including both ischemic and non-ischemic patterns of myocardial fibrosis. Moreover, our method resulted in the identification of the presence of the myocardial fibrosis for each LV AHA sector, thus contributing to a more precise regional analysis of the LV myocardium, as usually conducted in clinical practice. More recently, the ability to predict myocardial fibrosis from early CE-CCT was also demonstrated by integrating AI and radiomic features (28). Differently from our approach, only one slice for each sector of the heart (i.e., base, middle and apex) was analyzed, thus making it difficult to detect scar tissue in the acquired CCT volume and hampering the translation of the methodology into the actual clinical practice. This limitation was overcome in our work, with the classification of every slice in the CCT volume, followed by the integration of the results into a more conventional AHA 16-segments model that could help the clinician in focusing more the attention to those slices and segments suggested as pathological.

In line with previous studies, results suggest the potential role of AI-based technologies as a support for individual-level diagnosis into routine workflow, classifying myocardial sectors affected by fibrosis using early-enhanced CCT images, even when this is not clearly visible by human eyes. Besides the promising results, our study is not free from limitations. First, the image quality of the late CE-CCT, from which scar manual tracing was performed, is worse than that of the CE-CMR, thus potentially introducing under- or over-estimation in the scar size, which might explain the cause for false positives in CNN diagnoses. As our aim was to analyze all slices covering the LV included in the CCT volume, using scar manual tracing on CE-CMR would not have provided the proper reference, due to its limited number of slices. Accordingly, our choice of performing scar manual tracing on late CE-CCT, while having available as reference the CE-CMR images, was taken as an attempt to minimize the described above limitations. Second, considering that there is no consensus on the optimal time delay when to acquire late CE-CCT with sufficiently good image quality, and that only a single attempt should be performed to limit the exposure of patient to x-rays, the same time delay was applied in all the acquired patients. This setting could have limited the image quality in same patients, thus potentially affecting the manual segmentation accuracy, and raising the hypothesis that variability in performance between ischemic and non-ischemic patients might be likely related to image quality. Third, possible misalignment among early and late CE-CCT slices due to heart rate variation or different breath-holding needed to be adjusted when visually detected by manually re-setting the long-axis. In addition, this evaluation was performed as a single-center study using the same scanner for all the acquisitions, so further external evaluation (i.e., using different population and/or different scanner) is required to prove generalizability of our CNN model. Also, a larger training population could further improve the performance. Unfortunately, it is not common for patients to undergo both CMR and CCT in such a short window time, so specific research protocols need to be performed for this aim. Fourth, large volume of iodinated contrast-agent was used to account for the detection of myocardial delayed enhancement, thus potentially hampering the model’s performance at lower concentration of contrast agent. Finally, while scar location and scar tissue area lead to different prognoses (29), where the ratio of scar to LV myocardial mass represents an important factor for sudden cardiac death risk, the quantification of the myocardial fibrosis area was beyond our scope: only the indication of the possible LV myocardial segments affected by scar was given.

We addressed the challenging topic of myocardial fibrosis from routine noninvasive coronary scans. To this end, a novel AI system for myocardial characterization was proposed. The results suggest that the developed method has the potential to classify both ischemic and non-ischemic myocardial fibrosis sectors from early CE-CCT acquisition, thus removing the need of additional contrast-agent administration or radiations. This might potentially facilitate the investigation and management of patients with LV dysfunction and coronary artery disease. Further, being CE-CCT more widely available than CE-CMR, it could place CCT in a favorable position for a faster myocardial tissue characterization than CE-CMR.

## Data Availability

The datasets presented in this article are not readily available because of institutional policies. Requests to access the datasets should be directed to marco1.penso@mail.polimi.it.
