# Supplementary material for: A deep-learning approach for myocardial fibrosis detection in early contrast-enhanced cardiac CT images
Source: Front Cardiovasc Med. 2023 Jun 22;10:1151705. doi: 10.3389/fcvm.2023.1151705 (PMC10325686; doi:10.3389/fcvm.2023.1151705)
Supplement: Supplementary file 1 [file Table1.docx]

Supplementary Material

A Deep-Learning Approach for Myocardial Fibrosis Detection in Early Contrast-Enhanced Cardiac CT Images

Marco Penso^1,2*^, Mario Babbaro^3^, Sara Moccia^4^, Andrea Baggiano^1,5^, Maria Ludovica Carerj^1,6^, Marco Guglielmo^7,8^, Laura Fusini^1,2^, Saima Mushtaq^1^, Daniele Andreini^1,5^, Mauro Pepi^1^, Gianluca Pontone^1†^, Enrico G. Caiani^2,9†^

†These authors share last authorship

*** Correspondence:** Marco Penso: [marco1.penso@mail.polimi.it](mailto:marco1.penso@mail.polimi.it)

# Supplementary Methods

## Diagnostic criteria for cardiomyopathy

Diagnosis of ischemic dilated cardiomyopathy was based on the presence of an ischemic pattern of contrast-enhanced (CE) and following a coronary distribution. Non-ischemic dilated cardiomyopathy was considered in case of midwall septal CE distribution. Hypertrophic cardiomyopathy was defined by increased left ventricular (LV) wall thickness ≥15mm and in the absence of other cardiac or systemic diseases that could explain a similar hypertrophy. LV non-compaction cardiomyopathy was recognized on the basis of the diagnostic criteria previous described (1). Finally, the criteria to diagnose myocarditis was the presence of definite subepicardial CE distribution.

1. Authors/Task Force members; Elliott PM, Anastasakis A, Borger MA, Borggrefe M, Cecchi F, Charron P, Hagege AA, Lafont A, Limongelli G, Mahrholdt H, McKenna WJ, Mogensen J, Nihoyannopoulos P, Nistri S, Pieper PG, Pieske B, Rapezzi C, Rutten FH, Tillmanns C, Watkins H. 2014 ESC Guidelines on diagnosis and management of hypertrophic cardiomyopathy: the Task Force for the Diagnosis and Management of Hypertrophic Cardiomyopathy of the European Society of Cardiology (ESC). Eur Heart J. 2014 Oct 14;35(39):2733-79.

## AHA sectors

CCT images were processed in order to obtain myocardial sectors according to the division proposed by the American Heart Association (AHA) model. From the short axis, the 6 AHA-sectors division was based on the definition of two reference points: the central point of the ventricular cavity (C) and the point of insertion (PI) of the right ventricular wall to the LV, also called atrioventricular junction. For each image, the point C was automatically estimated as the centroid of the endocardial region while the PI was defined by the intersection of the line defined by taking the position of the atrio-ventricular junction in the first and last slice. Once PI and C were set in each image, the angle α was defined as included between the vertical line passing through C and the line joining C and PI. Starting from the reference point PI, moving clockwise of a defined angle, it is possible to identify the sectors.

Due to the larger number of slices in the CE-CCT volume compared to the CE- cardiac magnetic resonance (CMR) dataset, a sector in the early CE-CCT AHA model was considered as “scar” if the same sector was classified as “scar” by the CNN approach in at least eight slices in the same section (i.e., basal, mid, apical). This cut-off value was chosen empirically on the training set to minimize the classification error.

# Supplementary Tables

**Supplementary Table 1:** Pattern distribution of late gadolinium enhancement

| **Patient #** | **Segments model according to AHA (Type)** | **Sum** |
| --- | --- | --- |
| 1 | 5 (Sub-epi); 2, 3, 8, 9 (Mid-wall) | 5 |
| 2 | 10 (Sub-endo) | 1 |
| 3 | 2, 8, 9 (Mid-wall) | 3 |
| 4 | 2, 3 (Mid-wall) | 2 |
| 5 | 2-5, 10 (Mid-wall) | 5 |
| 6 | 4, 5 (Mid-wall) | 2 |
| 7 | 4, 5, 10, 11 (Trans); 3, 9 (Sub-endo) | 6 |
| 8 | 13, 14, 17 (Trans) | 3 |
| 9 | 2, 3 (Mid-wall); 10, 15 (Sub-endo) | 4 |
| 10 | 7, 8, 12 (Sub-endo); 13-17 (Trans) | 8 |
| 11 | 2, 3, 8, 9 (Mid-wall) | 4 |
| 12 | 5, 11 (Sub-endo); 2 (Mid-wall); 3 (Sub-epi) | 4 |
| 13 | 7-9 (Sub-endo); 13-17 (Trans) | 8 |
| 14 | 2, 7, 8, 13-15, 17 (Trans) | 7 |
| 15 | 2, 3, 5 (Sub-epi) | 3 |
| 16 | 5, 9 (Sub-endo); 3, 4, 10, 11 (Trans) | 6 |
| 17 | 2-5, 7-10, 13, 14 (Sub-epi) | 10 |
| 18 | 5, 8, 11 (Trans) | 3 |
| 19 | 2, 3 (Mid-wall); 5, 9, 14-17 (Trans) | 8 |
| 20 | 1-10, 13, 14 (Sub-epi) | 12 |
| 21 | 10, 15 (Trans) | 2 |
| 22 | 2, 3, 5, 8 (Mid-wall) | 4 |
| 23 | 5 (Mid-wall) | 1 |
| 24 | 2-4, 8, 9 (Mid-wall); 10, 11 (Sub-endo) | 7 |
| 25 | 2, 8, 11, 12, 14, 16 (Sub-endo); 1, 7, 13, 17 (Trans) | 10 |
| 26 | 1, 3, 7 (Sub-epi) | 3 |
| 27 | 4, 5 (Sub-epi) | 2 |
| 28 | 11 (Trans) | 1 |
| 29 | 1, 5, 6, 10-12, 16 (Sub-epi) | 7 |
| 30 | 4, 5 (Sub-endo); 10, 11, 15, 16 (Trans) | 6 |
| 31 | 1-3, 7-9, 14 (Sub-epi) | 7 |
| 32 | 2, 3, 8, 9 (Mid-wall) | 4 |
| 33 | 8 (Sub-epi) | 1 |
| 34 | 5 (Sub-epi); 8, 9 (Mid-wall) | 3 |
| 35 | 2-5, 8-11, 13-16 (Mid-wall) | 12 |
| 36 | 2, 3, 8, 9 (Mid-wall) | 4 |
| 37 | 8-10, 12, 14-16 (Sub-endo); 17 (Trans) | 8 |
| 38 | 5, 6, 14, 17 (Trans) | 4 |
| 39 | 2-5, 8-10 (Mid-wall) | 7 |
| 40 | 4 (Sub-epi) | 1 |
| 41 | 2, 3, 8, 9 (Sub-epi) | 4 |
| 42 | 2, 3, 8, 9 (Sub-epi) | 4 |
| 43 | 12 (Sub-endo); 8, 13-16 (Trans) | 6 |
| 44 | 2-5, 8, 9 (Mid-wall) | 6 |
| 45 | 2, 3 (Mid-wall) | 2 |
| 46 | 8, 9 (Mid-wall) | 2 |
| 47 | 5 (Mid-wall) | 1 |
| 48 | 2, 3, 5 (Sub-epi) | 3 |
| 49 | 2, 3, 9 (Mid-wall) | 3 |
| 50 | 2, 6-8, 12-17 (Trans) | 10 |

*Sub-endo, subendocardial; Mid, mid-wall; Sub-epi, subepicardial; Trans, transmural; AHA, American heart association.*

## Supplementary Figures


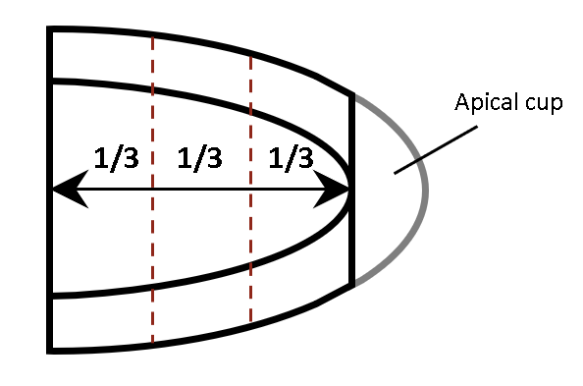


**Supplementary Figure 1.** Selection of cardiac slices. The left ventricle is divided into equal thirds perpendicular to the long axis of the heart: Basal, mid and apical; the apical cap, that is the area of myocardium beyond the end of the left ventricular cavity, was excluded by the apical section


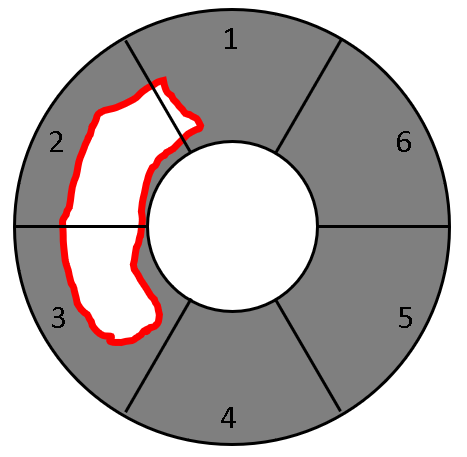


**Supplementary Figure 2.** Exclusion criteria. A very small (compared to the area of the myocardium) fibrotic region could be difficult to discriminate from a healthy region, therefore it was removed to reduce noise from datasets (train, validation and test set). The elimination criterion considers the percentage ratio between the scar and total myocardium area in a sector. Sectors completely free of fibrotic tissue were considered healthy, and those sectors with a ratio greater than 15% were considered pathological. These strict thresholds lead to the elimination of all sectors with small region of fibrotic tissue. Thus, if the ratio between scar area and myocardial area was in the interval (0%, 15%] the sector was discarded. Therefore, in the example, the sector 1 would have been excluded because it doesn’t match the condition previously reported.
